# Efficacy of different acupuncture therapies on hand dysfunction in post-stroke patients: a systematic review and meta-analysis

**DOI:** 10.3389/fneur.2025.1589874

**Published:** 2025-05-22

**Authors:** Yuxiang Liu, Jing Zhou, Jiawen Zheng, Jing Chen

**Affiliations:** ^1^The Third Clinical Medical College, Zhejiang Chinese Medical University, Hangzhou, China; ^2^Department of Rehabilitation, The Third Affiliated Hospital of Zhejiang Chinese Medical University, Hangzhou, Zhejiang, China

**Keywords:** acupuncture, electroacupuncture, hand dysfunction, stroke, meta-analysis

## Abstract

**Objective:**

Hand dysfunction is one of the main causes of disability in stroke. This systematic review and meta-analysis aimed to evaluate the efficacy of different types of acupuncture therapy in improving hand dysfunction among post-stroke patients.

**Methods:**

A comprehensive search was conducted across eight databases (PubMed, Cochrane Library, Embase, Web of Science, China National Knowledge Infrastructure [CNKI], Chongqing VIP Chinese Scientific Journals Database [VIP], China Biology Medicine [CBM], and Wan Fang) to identify randomized controlled trials (RCTs). Forty-two RCTs involving 2,766 participants were included. Primary outcomes were the Brunnstrom Recovery Stage (BRS), Fugl-Meyer Assessment (FMA), and Lindmark scores; secondary outcomes included the Modified Ashworth Scale (MAS), Range of Motion (ROM), Manual Muscle Testing (MMT), and Modified Barthel Index (MBI).

**Results:**

Meta-analyses demonstrated significant improvements in hand function across multiple outcomes: BRS (mean difference [MD] = 0.56, 95% confidence interval [CI]: 0.43–0.69), FMA (MD = 1.24, 95% CI: 0.96–1.53), MAS (MD = −0.48, 95% CI: −0.59 to −0.38), ROM (MD = 0.95, 95% CI: 0.64–1.26), and MBI (MD = 6.70, 95% CI: 4.85–8.55). Subgroup analyses revealed that electroacupuncture (EA) outperformed traditional acupuncture (TA) in improving BRS (*p* = 0.008). Heterogeneity was partially attributed to acupuncture modalities, with EA exhibiting lower variability compared to traditional methods.

**Conclusion:**

This meta-analysis supports the use of acupuncture, particularly EA, for enhancing hand function in post-stroke patients. EA demonstrates superior efficacy and consistency, suggesting its prioritization in clinical practice.

## 1 Introduction

Stroke is an acute cerebrovascular disease caused by cerebral vascular occlusion or rupture, leading to brain function disorder. It can be classified into ischemic stroke (1) or hemorrhagic stroke (2). Common sequalae of stroke include cognitive impairment, hemiplegia, aphasia, and other neurological deficits. Stroke is a prevalent condition, with 15 million cases occurring globally each year, ranking second in global mortality and third in disability rates (3). Hand dysfunction is a frequent complication of stroke and one of the main contributors to disability. Clinical manifestations include motor impairment, swelling, pain, and numbness in the affected hand, severely impacting activities of daily living. Additionally, it imposes significant psychological burdens (4). Moreover, the negative impact of hand dysfunction extends to increased caregiving costs for families and society (5, 6). Thus, identifying effective treatments for post-stroke hand dysfunction is imperative.

As a core component of traditional Chinese medicine, acupuncture is widely utilized in stroke rehabilitation. Its efficacy and cost-effectiveness have been demonstrated in multiple clinical trials. Substantial studies have confirmed the utility of acupuncture in improving various functional impairments in stroke patients (7, 8). Research further corroborated the definitive therapeutic effects on shoulder-hand syndrome (9) and hand spasticity in stroke patients (10). In recent years, the heightened awareness of rehabilitation in China has led to an increasing number of studies employing systematic rehabilitation assessments to evaluate acupuncture outcomes. This trend enables the execution of the present study. Building on prior research, we conducted a meta-analysis of clinical trials to assess the efficacy of acupuncture in treating post-stroke hand dysfunction, with subgroup analyses to identify factors contributing to variability in outcomes. Our objective is to evaluate the therapeutic effects of various acupuncture techniques on post-stroke hand function through the lens of modern rehabilitation theories, thereby providing higher-quality evidence for the significance of acupuncture in stroke rehabilitation. Additionally, we aim to offer novel insights into the selection of acupuncture modalities for patients with post-stroke hand dysfunction.

## 2 Data and methods

### 2.1 Search strategy

Two researchers independently reviewed randomized controlled trials (RCTs) published from January 1, 2005, to December 31, 2024. The search encompassed eight databases, including four English-language databases (Cochrane Library, PubMed, Embase, and Web of Science) and four Chinese-language databases (China National Knowledge Infrastructure [CNKI], Chongqing VIP Chinese Scientific Journals Database [VIP], China Biology Medicine [CBM], and Wan Fang Database). For PubMed, the search strategy employed the following:

MeSH terms and keywords: (((((“Stroke”[MeSH Terms]) OR (stroke[Title/Abstract])) OR (cerebrovascular accident[Title/Abstract])) OR (cerebral stroke[Title/Abstract])) OR (CVA[Title/Abstract])) AND (((“Acupuncture Therapy”[MeSH Terms]) OR (“Therapy, Acupuncture”[MeSH Terms])) OR (Acupuncture[Title/Abstract])) AND ((“Hand”[MeSH Terms]) OR (hand[Title/Abstract])).

Analogous search strategies were adapted for other databases. The complete search strategies for all databases are provided in Supplementary File 1.

### 2.2 Eligibility criteria

#### 2.2.1 Inclusion criteria

The inclusion criteria were as follows: (1) only RCTs investigating acupuncture for stroke treatment, published between January 1, 2005, and December 31, 2024, were included. (2) Participants exhibited hand dysfunction (regardless of age, sex, disease duration, stroke type, or treatment duration). (3) RCTs evaluating various acupuncture modalities for stroke were included. The distinction between experimental and healthy groups was that the experimental group received one additional acupuncture therapy compared to the healthy group. (4) Studies must report at least one primary outcome measure. Outcome Measures: Primary outcomes: Brunnstrom Recovery Staging (BRS) for hand, Fugl-Meyer Assessment (FMA) Upper Extremity wrist/hand items, Lindmark Hand Assessment. Secondary outcomes: Modified Ashworth Scale (MAS) for hand joints, Range of motion (ROM) of hand joints, Manual Muscle Testing (MMT) for hand musculature, and Modified Barthel Index (MBI).

#### 2.2.2 Exclusion criteria

The exclusion criteria were as follows: (1) including animal studies, observational studies, meta-analyses, reviews, and systematic reviews. (2) Studies with unclear or undefined diagnostic criteria. (3) Patients diagnosed with other condition/complication that affects hand function (e.g., shoulder-hand syndrome). (4) Studies lacking assessment methods for any primary outcome measures. (5) Studies where additional non-acupuncture therapies were introduced to the experimental group beyond the healthy group’s baseline treatment. (6) Duplicate publications of the same study across multiple language databases. (7) Studies with inaccessible full texts or insufficient data.

### 2.3 Data extraction and analysis

#### 2.3.1 Data extraction

Two researchers first excluded duplicate records using the deduplication function in EndNote X9, followed by screening titles and abstracts to remove irrelevant studies or duplicates that did not meet the inclusion criteria. Subsequently, full texts of the remaining studies were retrieved and independently assessed for eligibility based on the predefined inclusion and exclusion criteria. The researchers then checked their judgments. Any disagreement was resolved through discussion with a third researcher. A data extraction form was designed in Microsoft Excel to systematically extract the following information: publication year, country, first author, diagnostic criteria source, sample sizes of the experimental and healthy groups, demographic data (sex, age, disease duration), intervention methods for both groups, duration and frequency of interventions, outcome measures (primary and secondary), and adverse events.

#### 2.3.2 Quality assessment

The methodological quality of included studies was evaluated using the risk-of-bias tool integrated in RevMan 5.4 (Cochrane Collaboration), covering the following domains: (1) random sequence generation; (2) allocation concealment; (3) blinding of participants and personnel; (4) blinding of outcome assessors; (5) completeness of outcome data; (6) selective reporting; and (7) other potential biases. Each domain was classified as having a high risk, low risk, or unclear risk of bias (ROB). Any disagreements in assessment were resolved through discussion with a third investigator.

#### 2.3.3 Statistical analysis

Statistical analyses were conducted using RevMan 5.4. Outcome measures were converted to continuous data, and results were expressed as mean difference (MD) with 95% confidence intervals (CI). Heterogeneity was evaluated using the *p*-value and *I*^2^ statistic. A *p*-value ≥0.05 combined with *I*^2^ ≤ 50% indicated low statistical heterogeneity, whereas a *p*-value <0.05 and *I*^2^ > 50% suggested substantial heterogeneity. Subgroup analyses were subsequently performed to identify potential sources of heterogeneity. Forest plots and funnel plots were generated to assess the heterogeneity of meta-analysis results and publication bias, respectively. Publication bias was considered absent if the funnel plot exhibited symmetry; asymmetry indicated potential publication bias. RevMan 5.4 and Excel were utilized for the creation of graphs and tables.

## 3 Results

### 3.1 Study characteristics

**Figure 1:**
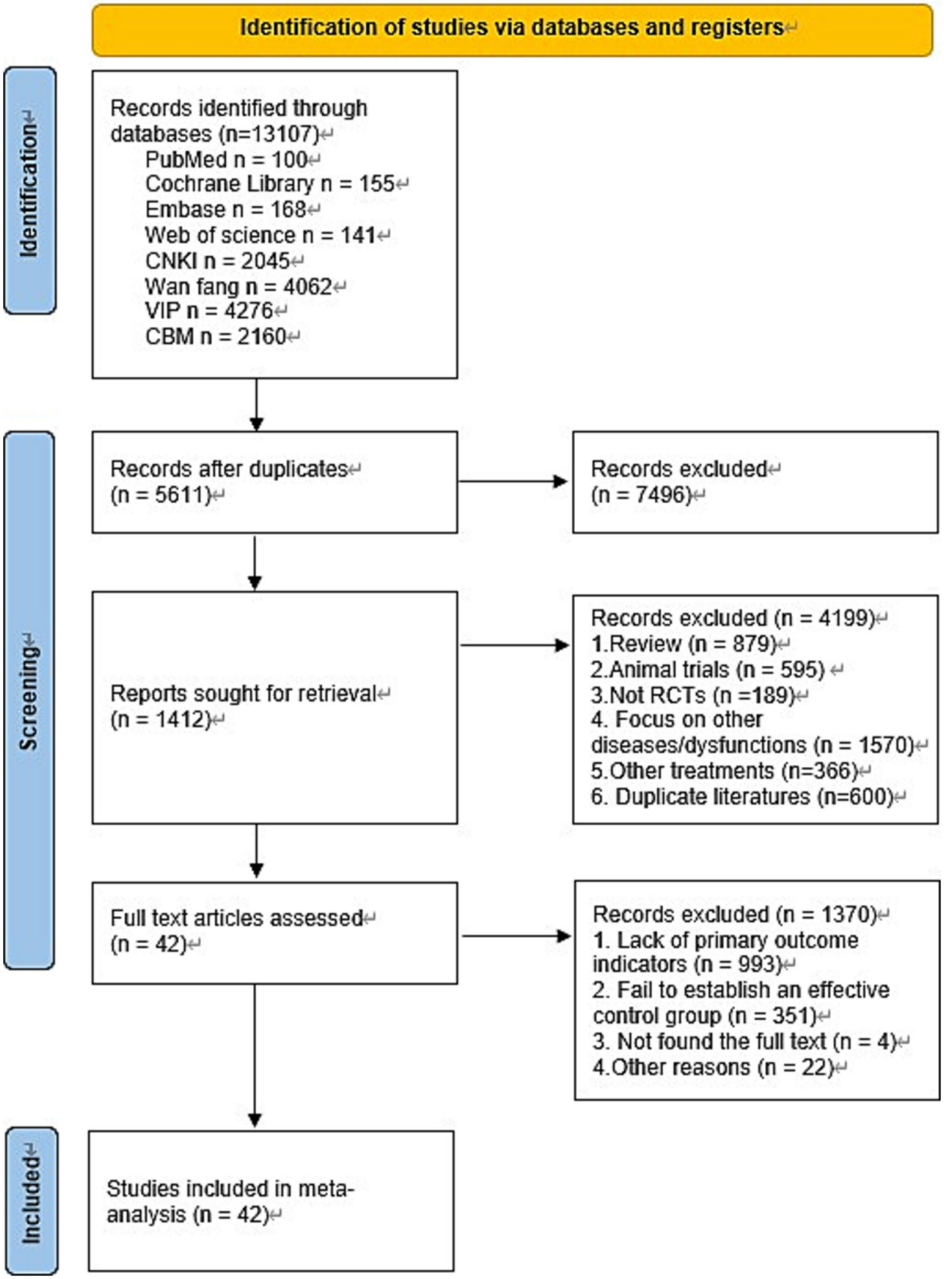
Flow diagram.

A total of 13,107 articles were identified through the search strategy: 100 from PubMed, 155 from Cochrane Library, 168 from Embase, 141 from Web of Science, 2,045 from CNKI, 4,062 from Wan fang, 4,276 from VIP, and 2,160 from CBM. After removing 7,496 duplicates, 4,199 articles were excluded based on title or abstract screening. The remaining 1,412 full-text articles underwent further review, and 42 studies were ultimately included. The detailed selection process is illustrated in Figure 1.

Table 1 summarizes the baseline characteristics of the included studies, detailing sample size, gender distribution, age and disease duration at baseline, intervention methods in experimental and healthy groups, stroke types, diagnostic criteria, outcome measures, and adverse events. A total of 42 studies involving 2,766 patients (1,380 in the experimental group and 1,386 in the healthy group) were included. Individual study sample sizes ranged from 22 to 120. Most studies reported gender distribution, with male participants predominating. The mean age of participants was reported separately for experimental and healthy groups in all studies except one, which provided combined data (11). Mean ages across studies ranged from 50 to 65 years. Disease duration was reported separately in 37 studies and combined in one study (11); five studies did not report disease duration. The mean disease duration varied widely, from 6 days to 32 months, meanwhile most studies reported durations between 2 weeks and 4 months. Twenty-four studies (12–35) included both ischemic and hemorrhagic stroke; 8 studies (36–43) enrolled ischemic stroke only, 10 studies (11, 44–52) did not specify stroke type.

**Table 1:** Characteristics of all included RCTs.

Trial Name	Sample size		Sex (male/female)		Age (years)		Disease course (months)		Methods of intervention		Extra acupuncture location	Therapy duration (weeks)	Type of stroke	Source of diagnostic criteria	Outcome measures	Adverse reactions
	T	C	T	C	T	C	T	C	T	C						
Huang Xinyun 2024 (12)	32	32	26/6	22/10	58.56 ± 11.85	60.25 ± 12.17	5.00 (3.25, 12.00)	6.00 (2.00, 11.00)	Traditional acupuncture	Acupuncture	Hand, arm, head and body	4	Ischemic or hemorrhage stroke	Not specified in detail	BRS, MAS, MMT, MBI	Not reported
Zhang Miao 2024 (36)	30	30	23/7	20/10	66.4 ± 8.4	69.4 ± 11.6	1.6 ± 1.1	1.7 ± 1.5	Traditional acupuncture	Acupuncture	Hand	4	Ischemic stroke	CT or MRI, Chinese expert consensus standards and guidelines	BRS, FMA, MAS, ROM	Not reported
Xie Qing 2024 (13)	30	30	23/7	19/11	60.27 ± 14.04	57.9 ± 8.91	Not reported	Not reported	Electroacupuncture	Rehabilitation + Functional electrical stimulation therapy	Hand, arm and head	4	Ischemic or hemorrhage stroke	Chinese expert consensus standards	BRS, FMA, MBI	Not reported
Liu Hong 2024 (14)	50	50	24/26	27/23	56 ± 5	57 ± 4	2.51 ± 0.98	2.15 ± 0.75	Warm Needling	Rehabilitation + Extracorporeal shock wave therapy	Hand, Arm, Head and Body	6	Ischemic or hemorrhage stroke	Chinese expert consensus standards	FMA	Not reported
Guo Bao 2024 (15)	51	51	27/24	29/22	56.17 ± 5.38	56.81 ± 5.41	2.83 ± 0.52	2.76 ± 0.51	Traditional acupuncture	Rehabilitation	Hand, arm and head	4	Ischemic or hemorrhage stroke	Chinese guidelines	BRS	Not reported
Wang Peixin 2023 (16)	30	30	17/13	18/12	52.30 ± 7.86	53.83 ± 13.05	0.842 ± 0.268	0.821 ± 0.277	Traditional acupuncture	Rehabilitation	Arm	4	Ischemic or hemorrhage stroke	CT/MRI, Chinese guidelines and instructional books	BRS, FMA, MBI	Not reported
Fan Sujin 2023 (44)	35	35	16/19	18/17	58.60 ± 7.23	58.77 ± 6.08	1.830 ± 0.345	1.801 ± 0.348	Traditional acupuncture	Acupuncture	Hand	4	Stroke	CT/MRI, Chinese guidelines	FMA, MAS	Not reported
Fan Dongqing 2023 (17)	30	33	16/14	17/16	56.90 ± 9.40	58.58 ± 10.56	3.07 ± 1.50	3.41 ± 1.80	Traditional acupuncture	Acupuncture	Hand	2	Ischemic or hemorrhage stroke	CT/MRI, Chinese guidelines	BRS, FMA, MAS, MBI	T: 1 case felt pain.
Zhao Fengfan 2022 (18)	30	30	18/12	14/16	62.9 ± 8.74	65.27 ± 9.51	3.24 ± 1.64	3.26 ± 1.35	Warm Needling	Rehabilitation	Hand	4	Ischemic or hemorrhage stroke	CT/MRI Chinese guidelines and instructional books	BRS, FMA	Not reported
Zhang Xinzhi 2022 (45)	25	25	11/14	12/13	65.16 ± 9.20	65.96 ± 8.98	3.34 ± 1.07	3.42 ± 1.04	Electroacupuncture	Rehabilitation	Hand and arm	3	Stroke	CT or MRI, Chinese expert consensus standards and guidelines	FMA, MAS, MBI	Not reported
Xu Weiyan 2022 (19)	30	30	18/12	16/14	60.47 ± 7.86	60.80 ± 8.56	2.73 ± 1.47	3.12 ± 1.50	Traditional acupuncture	Rehabilitation + Acupuncture	Hand	4	Ischemic or hemorrhage stroke	CT, Chinese guidelines	BRS, Lindmark, ROM, MBI	Not reported
Shao Yinjin 2022 (20)	42	43	24/18	20/23	54.14 ± 4.59	54.40 ± 7.28	2.16 ± 0.64	2.04 ± 0.69	Fire Needling	Rehabilitation	Arm	6	Ischemic or hemorrhage stroke	Chinese guidelines	FMA, MAS	Not reported
Long Xiaona 2022 (21)	30	31	18/12	17/14	63 ± 12	65 ± 9	0.733 ± 0.583	0.727 ± 0.522	Electroacupuncture	Rehabilitation + Rehabilitation Robot	Hand	6	Ischemic or hemorrhage stroke	Chinese guidelines	BRS, FMA, ROM	Not reported
Chen Ying 2022 (37)	32	32	17/15	18/14	57.25 ± 8.199	56.00 ± 8.436	2.85 ± 1.28	2.92 ± 1.08	Traditional acupuncture	Rehabilitation + Acupuncture	Hand	4	Ischemic stroke	Chinese guidelines	FMA, MAS	Not reported
Ma Nanda 2021 (38)	30	30	21/9	18/12	51.67 ± 12.430	55.23 ± 10.258	Not reported	Not reported	Traditional acupuncture	Rehabilitation	Hand, arm and head	4	Ischemic stroke	CT/MRI	BRS, MBI	Not reported
Jia Yanfei 2021 (46)	49	48	28/21	29/19	53.45 ± 6.60	52.65 ± 5.49	10.2 ± 1.92	9.96 ± 1.56	Electroacupuncture	Acupuncture	Arm	4	Stroke	Chinese expert consensus standards and guidelines	BRS, Lindmark	Not reported
Huang Linying 2021 (22)	30	30	15/15	18/12	60.63	58.87	Not reported	Not reported	Traditional acupuncture	Acupuncture	Hand + arm	5	Ischemic or hemorrhage stroke	Chinese guidelines	BRS, FMA, MAS	Not reported
Duan Yifei 2021 (39)	30	30	17/13	16/14	56.2 ± 9.4	55.3 ± 10.0	1.04 ± 0.32	1.09 ± 0.29	Traditional acupuncture	Rehabilitation	Hand + arm	8	Ischemic stroke	Chinese expert consensus standards	FMA, MAS, MBI	Not reported
Dou Jie 2021 (40)	47	47	29/18	27/20	58.53 ± 12.66	56.74 ± 14.90	31.38 ± 24.79	32.61 ± 19.46	Electroacupuncture	Rehabilitation + Acupuncture	Hand + arm	4	Ischemic stroke	CT/MRI, Chinese guidelines	BRS, MBI	Not reported
Wang Xiaochun 2020 (47)	33	34	18/15	16/18	51 ± 9	53 ± 10	2.16 ± 1.27	2.06 ± 1.09	Traditional acupuncture	Rehabilitation	Hand + arm	4	Stroke	CT/MRI, Chinese guidelines	FMA, MAS, MBI	T: 1 case felt pain.
Yuan Qin 2020 (23)	30	30	18/12	15/15	64.65 ± 2.51	65.34 ± 2.68	Not reported	Not reported	Traditional acupuncture	Rehabilitation + Acupuncture	Hand	4	Ischemic or hemorrhage stroke	CT/MRI, Chinese guidelines	FMA, MBI	Not reported
Ma Lifei 2020 (48)	20	20	12/8	11/9	61.85 ± 8. 28	60.45 ± 7. 08	1.45 ± 0. 68	1. 50 ± 0. 68	Traditional acupuncture	Rehabilitation	Hand and arm	4	Stroke	Chinese guidelines	BRS, MBI	Not reported
Liu Juan 2020 (24)	30	30	16/14	13/17	60.35 ± 6.20	61.67 ± 7.20	0.553 ± 0.253	0.549 ± 0.243	Fire needling	Acupuncture	Hand, arm and head	2	Ischemic or hemorrhage stroke	CT/MRI, Chinese expert consensus standards	BRS, MBI	Not reported
Zhang Xuan 2019 (25)	20	20	14/6	11/9	54.13 ± 12.73	55.21 ± 12.09	3.82 ± 2.16	3.78 ± 2.69	Traditional acupuncture	Rehabilitation	Hand and arm	4	Ischemic or hemorrhage stroke	CT/MRI, Chinese expert consensus standards	FMA, MBI	Not reported
Xian Zuxin 2019 (41)	43	43	23/20	26/17	65.27 ± 6.56	64.79 ± 6.48	2.79 ± 0. 54	2.83 ± 0.56	Electroacupuncture	Rehabilitation + Functional electrical stimulation therapy	Hand	6	Ischemic stroke	CT/MRI, Chinese guidelines	Lindmark, MBI	Not reported
Liu Jun’e 2019 (26)	60	60	33/17	35/25	60.60 ± 10.86	60.83 ± 9.62	2.77 ± 0.95	2.47 ± 0.94	Electroacupuncture	Rehabilitation	Hand and arm	10	Ischemic or hemorrhage stroke	Chinese expert consensus standards	FMA, MAS	Not reported
Ling Shanshan 2019 (42)	22	22	17/5	13/9	63.18 ± 9.93	57.77 ± 14.26	1.81 ± 1.57	2.197 ± 1.43	Traditional acupuncture	Rehabilitation	Body	4	Ischemic stroke	CT/MRI, Chinese instructional books	BRS, MBI	Not reported
Fan Hongyang 2019 (27)	30	30	19/11	19/11	61.3 ± 9.3	61.6 ± 9.6	3.7 ± 2.5	3.6 ± 2.4	Traditional acupuncture	Acupuncture	Hand	2	Ischemic or hemorrhage stroke	CT/MRI, Chinese guidelines and instructional books	BRS, FMA, MAS	Not reported
Chen Siqi 2018 (28)	30	31	16/14	17/14	59 ± 7	58 ± 6	3.91 ± 1.44	3.31 ± 1.17	Traditional acupuncture	Rehabilitation	Hand	9	Ischemic or hemorrhage stroke	Chinese expert consensus standards	FMA, MAS	T: 4 cases felt pain.
Weng Yilin 2018 (29)	29	27	18/11	15/12	56.90 ± 10.88	55.67 ± 10.57	0.97 ± 0.27	1.05 ± 0.19	Electroacupuncture	Rehabilitation	Arm	4	Ischemic or hemorrhage stroke	American and Chinese guidelines	FMA, MBI	Not reported
Sun Dingjiong 2018 (49)	25	25	11/9	8/12	63.27 ± 8.12	62.09 ± 7.89	0.41 ± 0.18	0.47 ± 0.17	Traditional acupuncture	Rehabilitation	Hand, arm and head	6	Stroke	CT/MRI, Chinese guidelines	FMA, MBI	Not reported
Tian Meng 2017 (30)	30	30	16/14	17/13	50.2 ± 4.6	49.9 ± 4.0	0.67 ± 0.14	0.63 ± 0.11	Electroacupuncture	Rehabilitation + Acupuncture	Hand	12	Ischemic or hemorrhage stroke	Chinese expert consensus standards	FMA, MAS, MBI	Not reported
Zhang Rui 2017 (31)	20	20	16/4	18/2	55.15 ± 10.97	54.90 ± 11.29	0.71 ± 0.72	1.11 ± 1.03	Traditional acupuncture	Rehabilitation+Mirror Therapy	Head	4	Ischemic or hemorrhage stroke	CT/MRI, Chinese expert consensus standards	BRS	Not reported
Wang Jie 2016 (50)	43	43	26/17	23/20	50.55 ± 12.99	51.45 ± 10.48	0.18 ± 0.11	0.16 ± 0.14	Traditional acupuncture	Rehabilitation	Hand	4	Stroke	CT/MRI, Chinese expert consensus standards	FMA, MBI	Not reported
Tulunayi Wanli 2016 (32)	45	45	22/23	13/21	56.7 ± 4.5	56.2 ± 4.1	1.58 ± 0.39	1.55 ± 0.35	Traditional acupuncture	Rehabilitation	Hand, arm and head	6	Ischemic or hemorrhage stroke	CT/MRI, Chinese expert consensus standards	FMA, MBI	Not reported
Yang Jiangxia 2015 (51)	40	40	27/13	25/15	60 ± 9	61 ± 9	2.52 ± 0.77	2.49 ± 0.71	Floating Needle Technique	Rehabilitation	Arm	8	Cerebrovascular disease	Chinese instructional books	FMA, MAS, MBI	Not reported
Jin Yisi 2015 (52)	18	18	9/9	10/8	57.81 ± 2.12	58.72 ± 3.14	0.261 ± 0.020	0.257 ± 0.017	Electroacupuncture	Rehabilitation	Arm	3	Stroke	Chinese guidelines	BRS	Not reported
Cui Shaoyang 2015 (33)	32	32	19/13	18/14	51.19 ± 7.89	52.28 ± 7.39	0.91 ± 0.19	0.94 ± 0.21	Traditional acupuncture	Rehabilitation + Mirror therapy	Hand, arm and head	4	Ischemic or hemorrhage stroke	CT/MRI, Chinese expert consensus standards	BRS, MBI	Not reported
Jiang Zaiyi 2014 (34)	11	11	Not reported	Not reported	55.55 ± 8.98	52.73 ± 14.55	1.70 ± 3.21	0.715 ± 1.01	Electroacupuncture	Rehabilitation	Hand, arm and head	2	Ischemic or hemorrhage stroke	CT/MRI, Chinese expert consensus standards	FMA	Not reported
Zhou Yu 2013 (35)	32	35	19/13	21/14	55.67 ± 8.75	57.09 ± 9.02	0.205 ± 0.048	0.197 ± 0.052	Traditional acupuncture	Rehabilitation	Head and hand	4	Ischemic or hemorrhage stroke	CT/MRI, Chinese expert consensus standards	FMA, MBI	Not reported
Zhuang Lixing 2011 (43)	44	43	23/21	24/19	65.47 ± 6.33	63.53 ± 7.35	1.45 ± 0.21	1.31 ± 0.24	Traditional Acupuncture	Rehabilitation	Hand, arm and head	5	Ischemic stroke	CT/MRI, Chinese expert consensus standards	BRS	C: 1 case was hematoma.
Chen Anliang 2008 (11)	30	30	Not reported	Not reported	Not specified in detail	Not specified in detail	Not specified in detail	Not specified in detail	Traditional Acupuncture	Rehabilitation	Hand and arm	4	Stroke	CT/MRI, Chinese expert consensus standards	Lindmark, MBI	Not reported

BRS, Brunnstrom Recovery stage (hand); FMA, Fugl-Meyer assessment (hand); Lindmark, Lindmark assessment (hand); MAS, Modified Ashworth Scale Assessment; ROM, range of motion; MBI, Modified Barthel Index. C, control group; T, test group.

In this study, the healthy groups of 8 studies (12, 17, 22, 24, 27, 36, 44, 46) utilized acupuncture therapy, while 23 studies (11, 15, 16, 18, 20, 25, 26, 28, 29, 32, 34, 35, 38, 39, 42, 43, 45, 47–52) employed conventional rehabilitation therapy in their healthy groups. The healthy groups of 5 studies (19, 23, 30, 37, 40) adopted acupuncture combined with conventional rehabilitation therapy, and 6 studies (13, 14, 21, 31, 33, 41) implemented conventional rehabilitation therapy integrated with novel rehabilitation methods. One study (42) administered sham acupuncture combined with rehabilitation therapy in the healthy group. Building upon the healthy group interventions, 26 studies (11, 12, 15–17, 19, 22, 23, 25, 27, 28, 31–33, 35–39, 42–44, 47–50) incorporated traditional acupuncture (TA) as an additional intervention, 11 studies (13, 21, 26, 29, 30, 34, 40, 41, 45, 46, 52) utilized EA, 2 studies (14, 18) applied warm acupuncture, 2 studies (20, 24) implemented fire acupuncture, and 1 study (51) adopted floating acupuncture. Adverse events were reported in 4 studies: two studies (17, 47) documented one case of pain in the experimental group, one study (28) reported four cases of pain in the experimental group, and one study (43) observed one case of subcutaneous hematoma in the healthy group.

### 3.2 Risk of bias

#### 3.2.1 Random sequence generation

Random sequence generation was described in 39 studies, among which 32 studies were assessed as low ROB. Specifically, 25 studies (13–17, 20, 24, 25, 27, 30, 31, 33, 34, 36, 39, 41, 42, 44–46, 48–52) utilized random number tables for sequence generation, while 7 studies (12, 22, 28, 29, 37, 43, 47) employed computer-generated randomization. Seven studies (11, 18, 21, 23, 26, 32, 38) were classified as unclear ROB due to insufficient methodological details, as they mentioned randomization without specifying the generation method. Three studies (19, 35, 40) were deemed high ROB because they assigned participants based on chronological order of enrollment.

#### 3.2.2 Allocation concealment

Three studies (12, 43, 47) implemented allocation concealment using sealed envelopes, and one study (30) mentioned allocation concealment without specifying the methodology. These studies were evaluated as low ROB. The remaining 38 studies (11, 13–29, 31–42, 44–46, 48–52) ignored the description of allocation concealment and were thus classified as unclear ROB.

#### 3.2.3 Blinding of participants and personnel

One study (12) adopted a double-blind design, and five studies (16, 17, 29, 36, 47) utilized a single-blind design; these were assessed as low ROB. The remaining 36 studies (11, 13–15, 18–28, 30–35, 37–46, 48–52) did not report the application of blinding for participants or personnel, resulting in an unclear ROB classification.

#### 3.2.4 Blinding of outcome assessment

Two studies (12, 29) applied blinding to outcome assessment and were therefore rated as low ROB. The remaining 40 studies did not describe the implementation of blinding for outcome assessment and were consequently deemed unclear ROB.

#### 3.2.5 Completeness of outcome data

Nine studies (12, 17, 20, 25, 28, 29, 37, 44, 47) explicitly reported dropouts and provided reasons for attrition or described methods for handling missing data. Thirty studies (11, 13–15, 18, 19, 21–24, 26, 27, 30–35, 38–43, 46, 48–52) documented no participant withdrawals during the trial. These studies were assessed as low ROB. Two studies (16, 45) reported dropouts but failed to clarify the reasons or data management strategies, and one study (36) exhibited significant data entry errors in one outcome measure; these were classified as high ROB.

#### 3.2.6 Selective reporting

All included studies reported all predefined outcome measures. Five studies (11, 13, 22, 23, 38) were deemed high ROB due to failure to separately report the disease course across intervention groups. The remaining 37 studies (12, 14–21, 24–37, 39–52) were evaluated as low ROB.

#### 3.2.7 Other risks of bias

**Figure 2:**
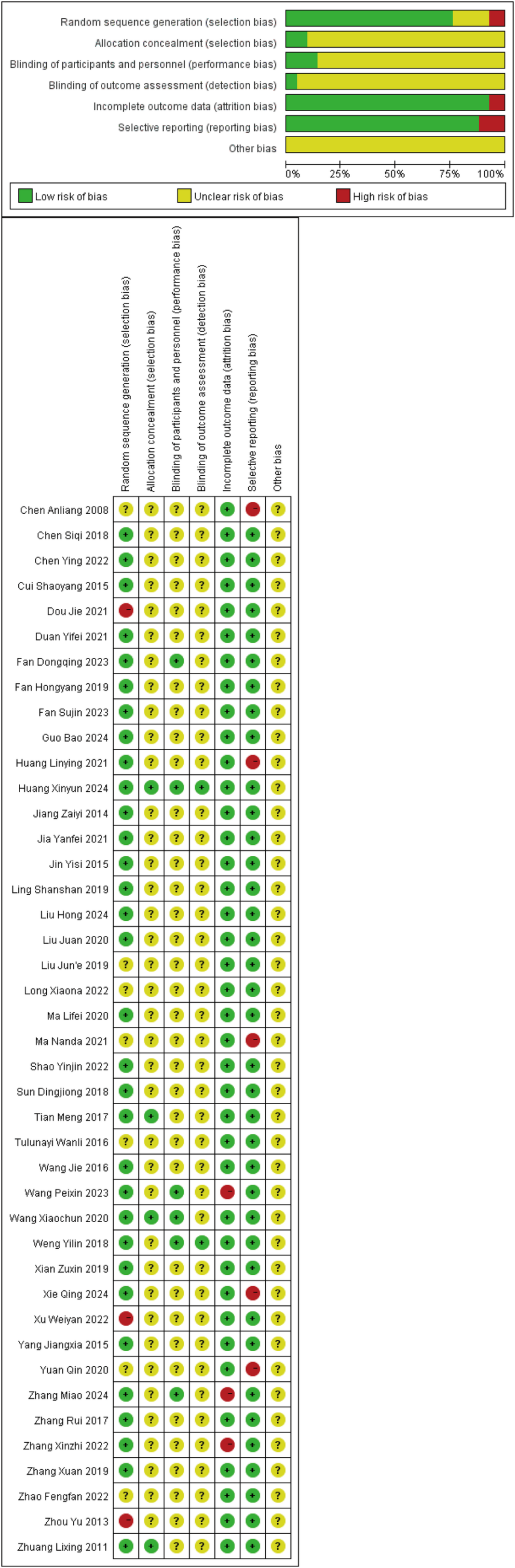
Risk of bias graph.

No studies reported information related to other sources of bias, resulting in an unclear ROB classification. Potential language bias may exist as only studies published in Chinese or English were included. Additionally, the predominance of studies conducted in China introduces potential geographical bias. ROB graph results are shown in Figure 2.

### 3.3 Meta-analysis of results

#### 3.3.1 Brunnstrom recovery stage (BRS)

A total of 21 studies (12, 13, 15–19, 21, 22, 24, 27, 31, 33, 36, 38, 40, 42, 43, 46, 48, 52) reported BRS as an outcome measure, involving 1,329 patients (664 in the experimental group and 665 in the healthy group). The random-effects meta-analysis demonstrated a significant improvement in hand BRS with acupuncture (MD = 0.56, 95% CI = 0.43–0.69, Chi^2^ = 45.12, *p* = 0.001, *I*^2^ = 56%), indicating that acupuncture effectively improves the BRS of the hand in stroke patients.

**Figure 3:**
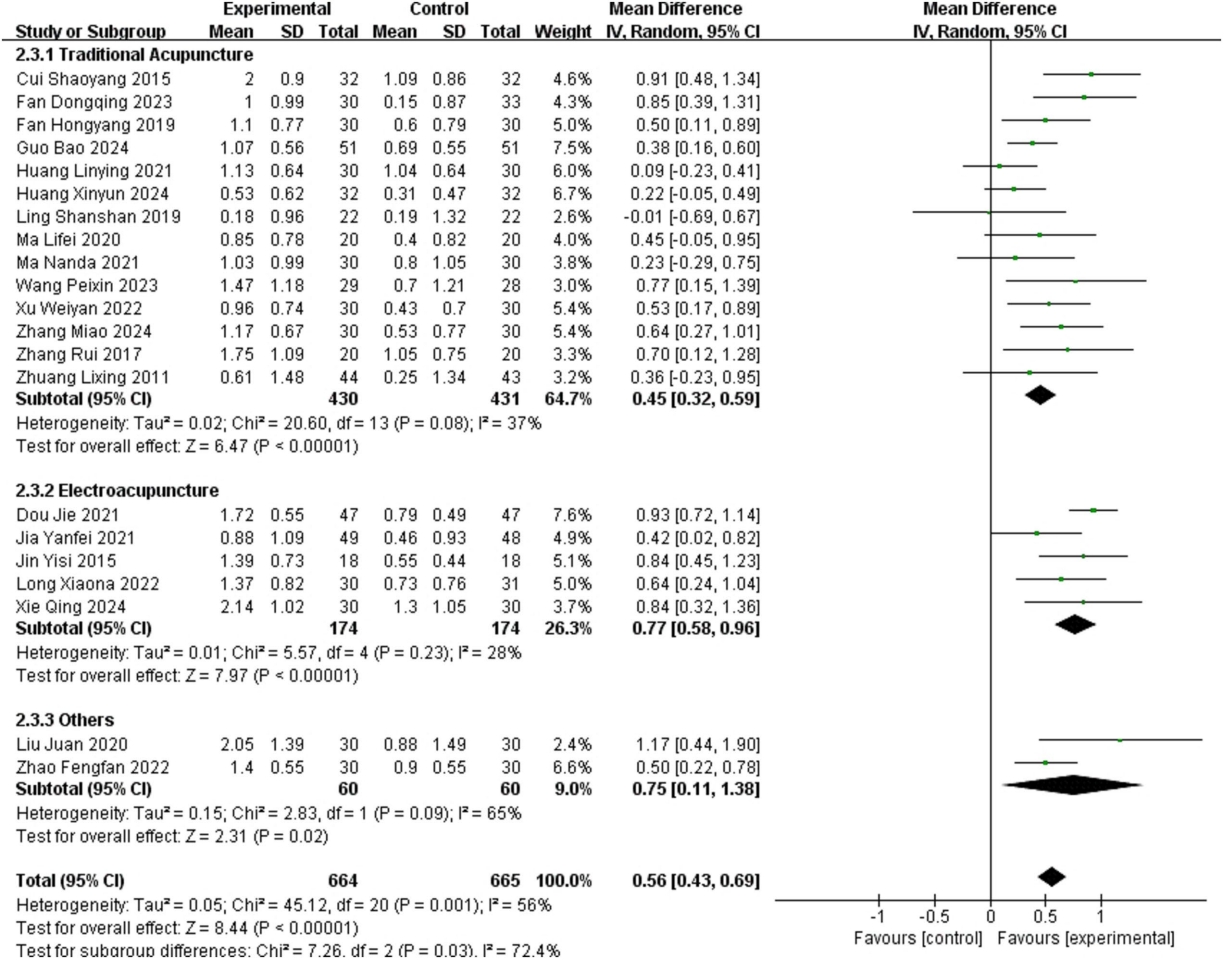
Meta-analysis of the effects of different acupuncture methods on BRS.

Analysis of 14 studies employing TA showed low within-subgroup heterogeneity (MD = 0.45, 95% CI = 0.32–0.59; *p* = 0.08, *I*^2^ = 37%). Similarly, the EA subgroup exhibited low heterogeneity (MD = 0.77, 95% CI = 0.58–0.96; *p* = 0.23, *I*^2^ = 28%). Subgroup analysis of two studies utilizing other acupuncture methods demonstrated high heterogeneity (MD = 0.75, 95% CI = 0.11–1.38; *p* = 0.09, *I*^2^ = 65%). These findings suggest that the choice of different acupuncture methods contributed to the heterogeneity. Details are presented in Figure 3.

The subgroup analysis explained heterogeneity in BRS. Significant differences in effect sizes were observed between subgroups (Chi^2^ = 7.26, *p* = 0.03, *I*^2^ = 72.4%), whereas within-subgroup heterogeneity was negligible. Pairwise comparisons identified statistically significant differences between TA and EA subgroups (Chi^2^ = 6.95, *p* = 0.008 [<0.0167], *I*^2^ = 85.6%), with no significant differences in other comparisons. This suggests distinct effects of TA and EA on BRS. Bonferroni correction is shown in Supplementary File 2.

#### 3.3.2 Fugl-Meyer assessment (FMA)

A total of 27 studies (13, 14, 16–18, 20–23, 25–30, 32, 34–37, 39, 44, 45, 47, 49–51) reported FMA as an outcome measure, involving 1,769 patients (881 in the experimental group and 888 in the healthy group). The random-effects meta-analysis revealed a large effect size favoring acupuncture (SMD = 1.24, 95% CI = 0.96–1.53, Chi^2^ = 189.71, *p* < 0.001, *I*^2^ = 86%).

**Figure 4:**
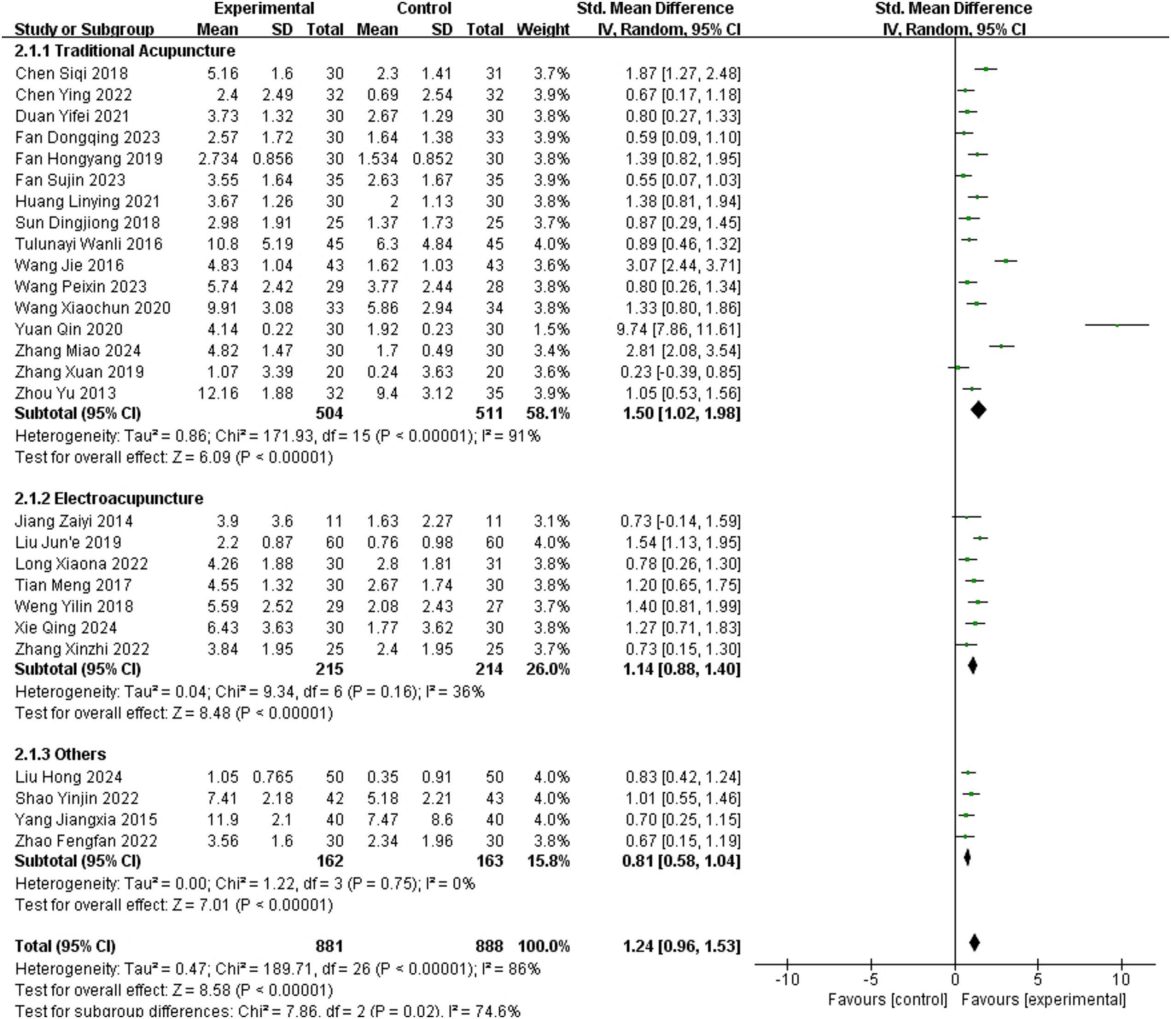
Meta-analysis of the effects of different acupuncture methods on FMA.

Subgroup analysis of 16 studies employing TA demonstrated significant within-subgroup heterogeneity (SMD = 1.50, 95% CI = 1.02–1.98; *p* < 0.001, *I*^2^ = 91%). In contrast, subgroup analysis of 7 studies using EA exhibited low heterogeneity (SMD = 1.14, 95% CI = 0.88–1.40; *p* = 0.16, *I*^2^ = 36%). Subgroup analysis of 4 studies utilizing other acupuncture methods also showed low heterogeneity (SMD = 0.81, 95% CI = 0.58–1.04; *p* = 0.75, *I*^2^ = 0%). It suggested that heterogeneity differences between subgroups may be associated with acupuncture methods. Details are presented in Figure 4.

The subgroup analysis partially explained the heterogeneity in FMA. Significant differences in effect sizes were observed between subgroups (Chi^2^ = 7.86, *p* = 0.02, *I*^2^ = 74.6%). Pairwise comparisons indicated potential statistical differences primarily between the TA and other acupuncture methods subgroups. However, due to the diversity of techniques categorized under “others” and the study’s primary focus on comparing TA with EA, no further analyses were conducted. After excluding a study in which potential data – entry errors might have occurred (36), the research findings of this part remained unaffected.

#### 3.3.3 Lindmark

Four studies (11, 19, 41, 46) reported the Lindmark as an outcome measure, involving 303 patients (152 in the experimental group and 151 in the healthy group). A random-effects model showed that acupuncture was equally effective in improving Lindmark scores (SMD = 1.34, 95 %CI = 0.38–2.30, Chi^2^ = 41.43, *p* < 0.001, *I*^2^ = 93%).

Analysis of 2 studies employing TA showed a significant improvement in hand Lindmark scores (SMD = 1.21, 95% CI = 0.09–2.33; *p* = 0.005, *I*^2^ = 87%). In contrast, subgroup analysis of 2 studies using EA revealed inconclusive effects on Lindmark scores (SMD = 1.48, 95% CI = −0.50–3.46; *p* < 0.001, *I*^2^ = 97%).

**Figure 5:**
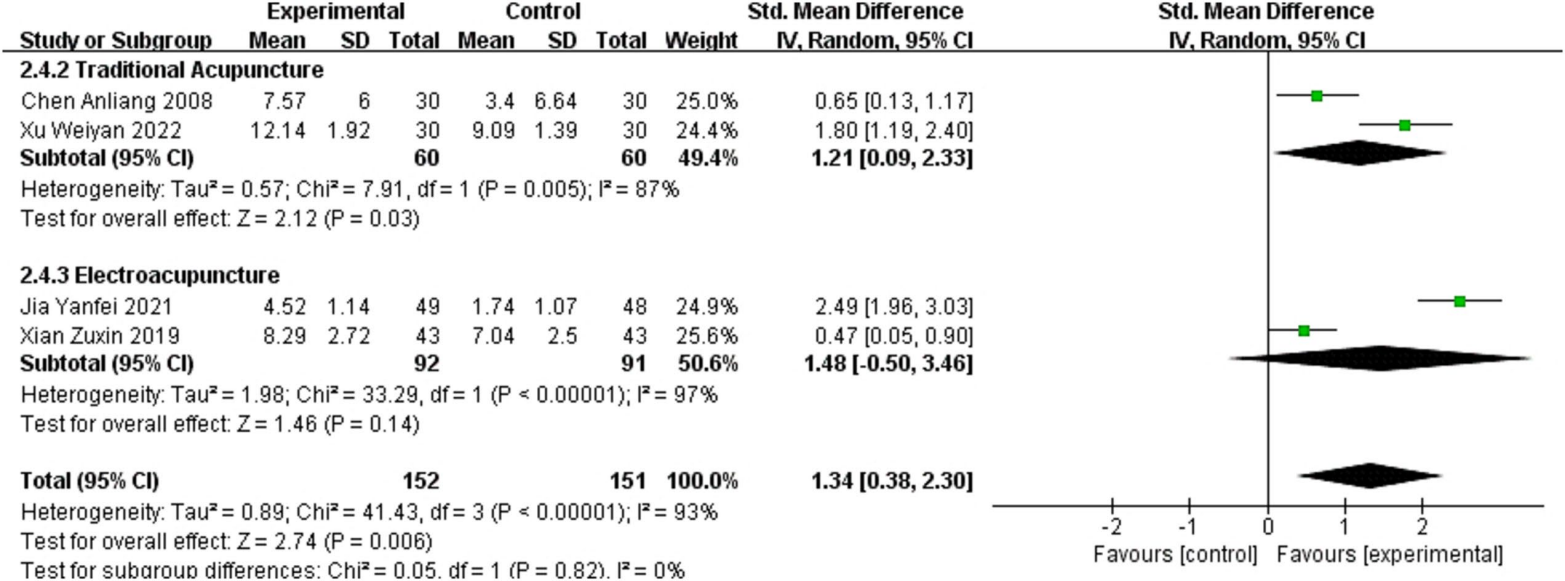
Meta-analysis of the effects of different acupuncture methods on Lindmark scores.

Differences in effect sizes between subgroups were not statistically significant (Chi^2^ = 0.05, *p* = 0.82, *I*^2^ = 0%). However, due to the limited number of included studies and substantial variability among them, these findings should be interpreted with caution. Details are presented in Figure 5.

#### 3.3.4 Modified Ashworth Scale (MAS)

Fifteen studies (12, 17, 20, 22, 26–28, 30, 36, 37, 39, 44, 45, 47, 51) reported MAS as an outcome measure, involving 1,024 patients (509 in the experimental group and 515 in the healthy group). The fixed – effect model indicates that acupuncture was effective in improving MAS scores in stroke patients, showing excellent consistency (MD = −0.48, 95% CI = −0.59–0.38, Chi^2^ = 16.13, *p* = 0.31, *I*^2^ = 13%).

Analysis of 10 studies using TA showed low within-subgroup heterogeneity (MD = −0.50, 95% CI = −0.63–0.37; *p* = 0.17, *I*^2^ = 30%). Subgroup analysis of 3 studies applying EA demonstrated negligible heterogeneity (MD = −0.60, 95% CI = −0.83–0.37; *p* = 0.98, *I*^2^ = 0%). Subgroup analysis of 2 studies utilizing other acupuncture methods suggested high heterogeneity (MD = −0.31, 95% CI = −0.55–0.07; *p* = 0.58, *I*^2^ = 65%).

**Figure 6:**
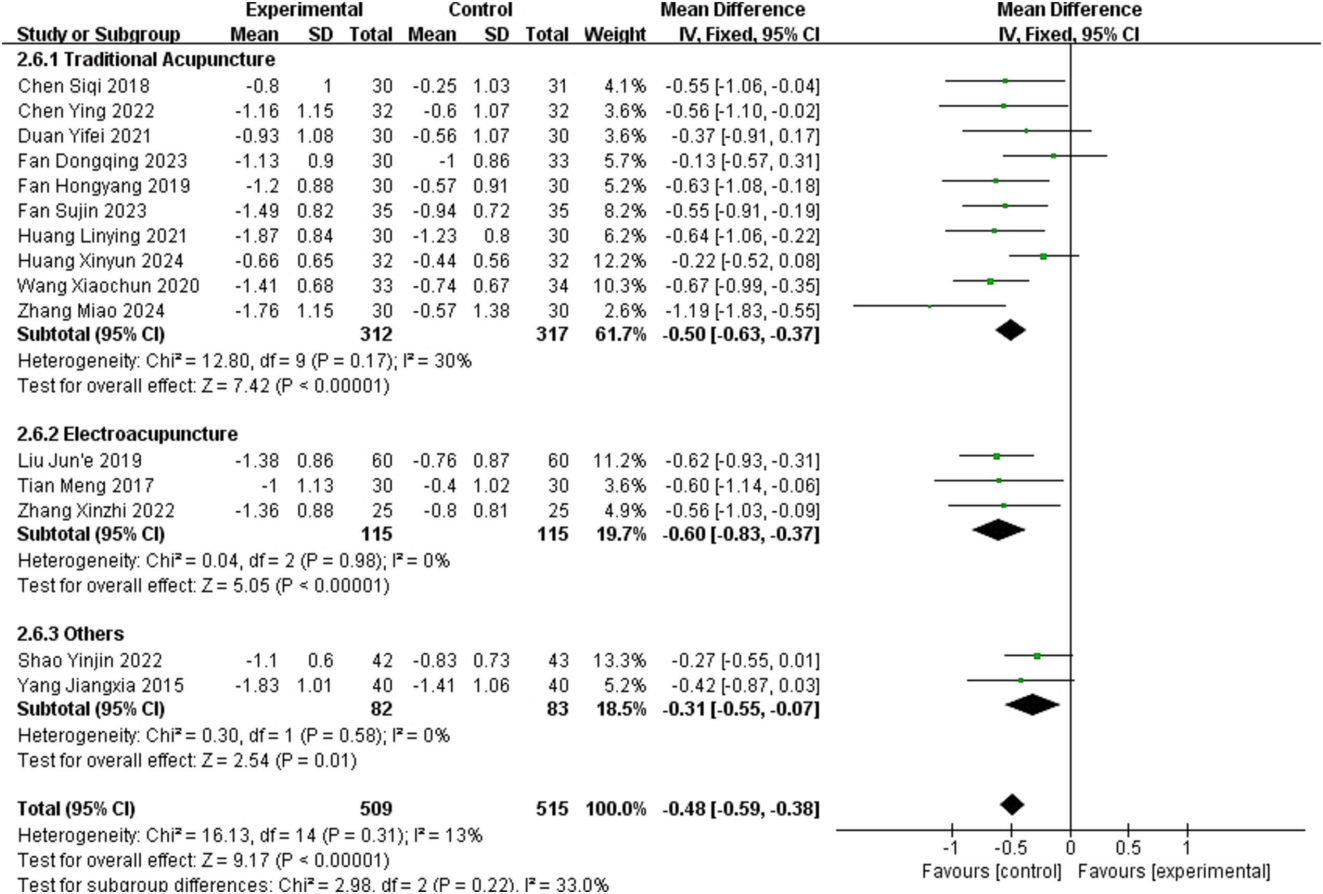
Meta-analysis of the effects of different acupuncture methods on MAS.

No significant differences in effect sizes were observed between subgroups (Chi^2^ = 2.98, *p* = 0.22, *I*^2^ = 33.0%), implying comparable efficacy among different acupuncture regimens in improving hand MAS grades in stroke patients. Details are presented in Figure 6.

#### 3.3.5 Range of motion (ROM)

Three studies (19, 21, 36) reported ROM as an outcome measure, involving 181 patients (90 in the experimental group and 91 in the healthy group). Pooled data from the meta-analysis demonstrated that acupuncture improved hand ROM in stroke patients (SMD = 0.95, 95% CI = 0.64–1.26; *p* = 0.71, *I*^2^ = 0%).

**Figure 7:**
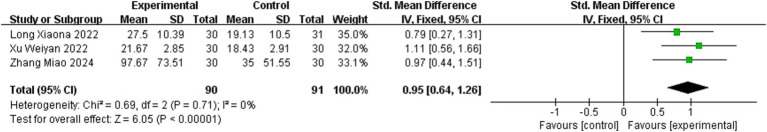
Meta-analysis of the effects of different acupuncture methods on ROM.

Subgroup analysis was not performed due to the limited number of studies and the unambiguous effect size. Details are presented in Figure 7.

#### 3.3.6 Modified Barthel Index (MBI)

Twenty-five studies (11–13, 16, 17, 19, 23–25, 29, 30, 32, 33, 35, 38–42, 45, 47–51) reported MBI as an outcome measure, involving 1,578 patients (787 in the experimental group and 791 in the healthy group). The random – effects model demonstrated the effectiveness of acupuncture, accompanied by substantial heterogeneity (MD = 6.70, 95% CI = 4.85–8.55, Chi^2^ = 256.23, *p* < 0.001, *I*^2^ = 91%).

**Figure 8:**
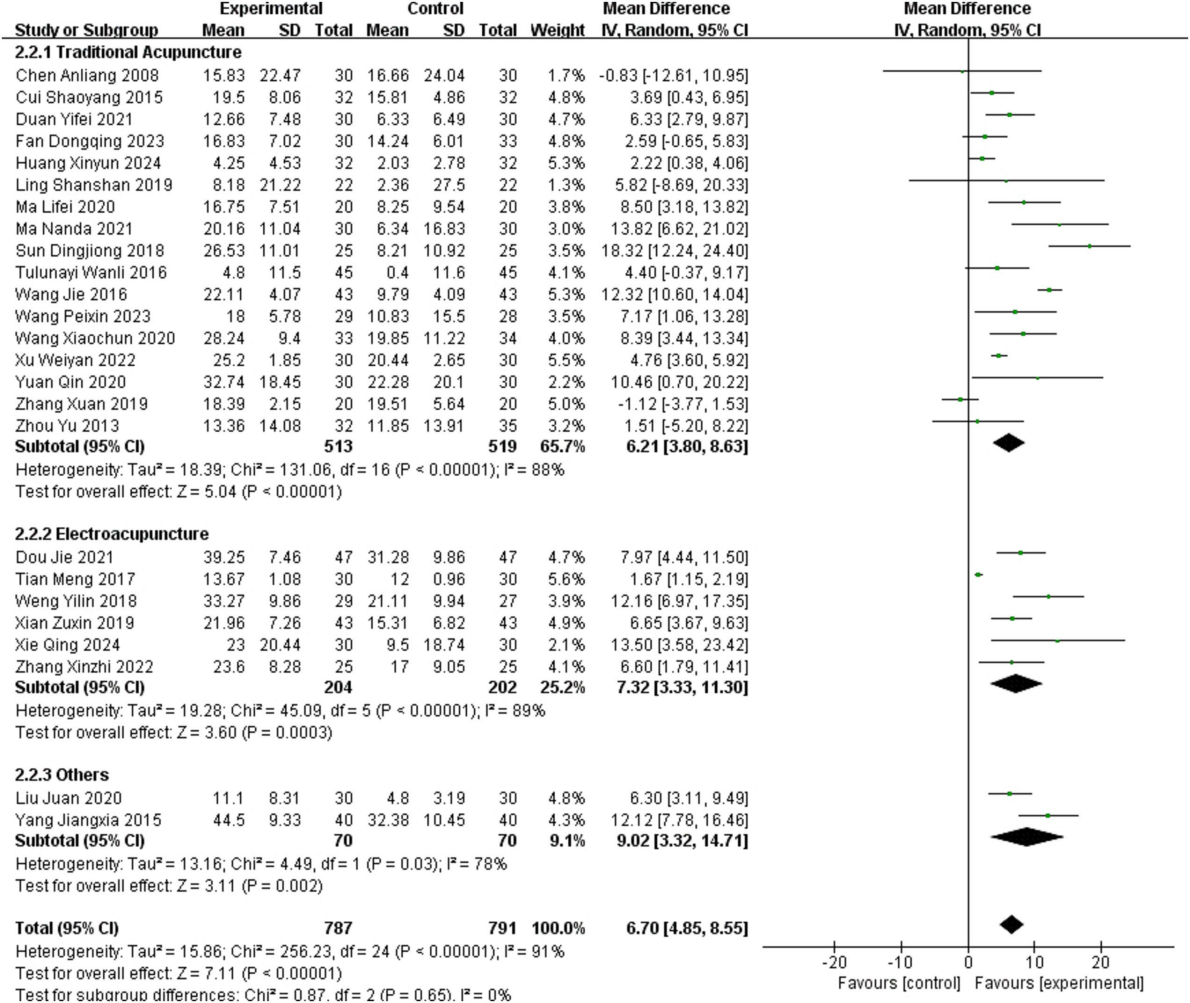
Meta-analysis of the effects of different acupuncture methods on MBI.

Subgroup analysis based on acupuncture methods was performed using a random-effects model. The analysis showed high heterogeneity within the TA subgroup (MD = 6.21, 95% CI = 3.80–8.63; *p* < 0.001, *I*^2^ = 88%). Similarly, EA subgroup exhibited substantial heterogeneity (MD = 7.32, 95% CI = 3.33–11.30; *p* < 0.001, *I*^2^ = 89%). The two studies employing other acupuncture methods also demonstrated high heterogeneity (MD = 9.02, 95% CI = 3.32–14.71; *p* = 0.03, *I*^2^ = 78%). Further details are provided in Figure 8.

**Figure 9:**
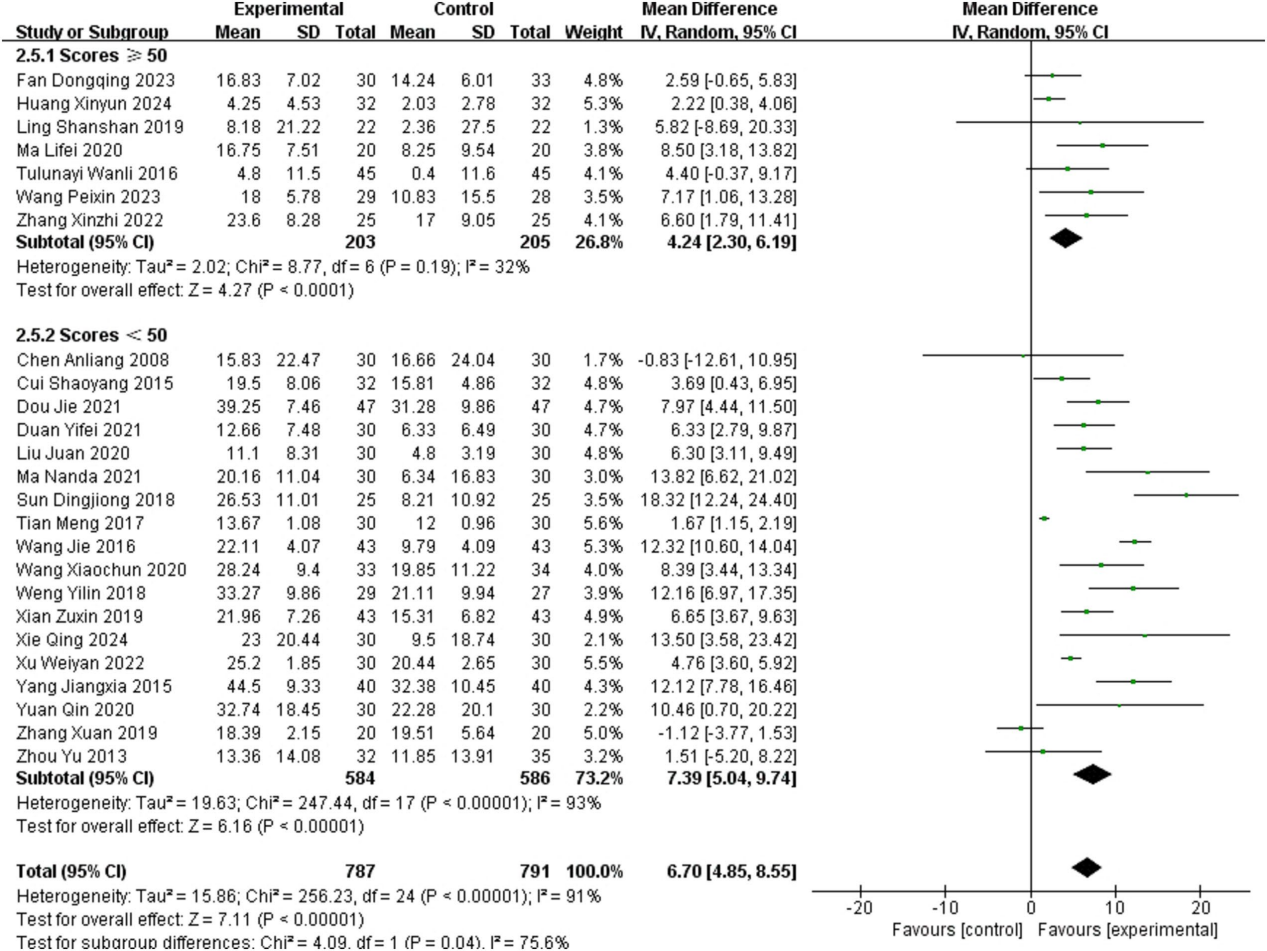
Meta-analysis of acupuncture effects on the MBI stratified by baseline scores.

As subgroup analyses stratified by acupuncture methods failed to account for the heterogeneity in the Modified Barthel Index (MBI), a secondary subgroup analysis was performed by categorizing studies into two groups using a baseline MBI score of 50 as the threshold. For studies involving patients with baseline MBI scores ≥50, the subgroup exhibited low heterogeneity (MD = 4.24, 95% confidence interval CI = 2.30–6.19; *p* = 0.19, *I*^2^ = 32%). In contrast, significant heterogeneity persisted in the subgroup with baseline MBI scores <50 (MD = 7.39, 95% CI = 5.04–9.74; *p* < 0.001, *I*^2^ = 93%). Subgroup analysis based on MBI scores partially explained the heterogeneity in acupuncture’s effects on MBI outcomes among stroke patients. Compared with patients with high MBI scores, acupuncture might have a more significant effect on patients with low MBI scores (Chi^2^ = 4.09, *p* = 0.04 [>0.025], *I*^2^ = 75.6%). Further details are available in Figure 9.

#### 3.3.7 Manual Muscle Testing (MMT)

Only one study (12) compared the MMT scores between the experimental and healthy groups, reporting no statistically significant effect (*p* = 0.311). Consequently, subgroup analysis was not performed due to insufficient data.

#### 3.3.8 Sensitivity analysis

**Figure 10:**
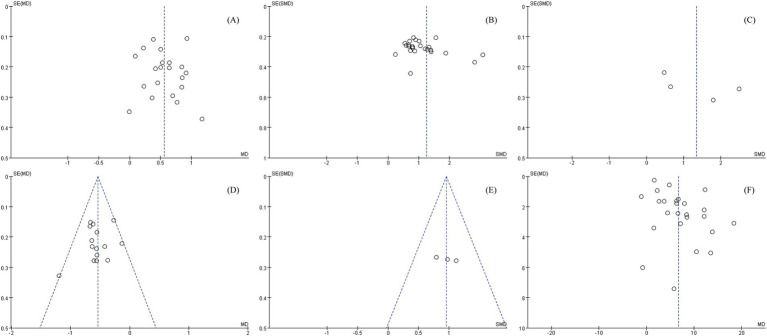
Sensitivity analyses of various outcome indicators, **(A)** BRS, **(B)** FMA, **(C)** Lindmark, **(D)** MAS, **(E)** ROM, **(F)** MBI.

**Figure 11:**
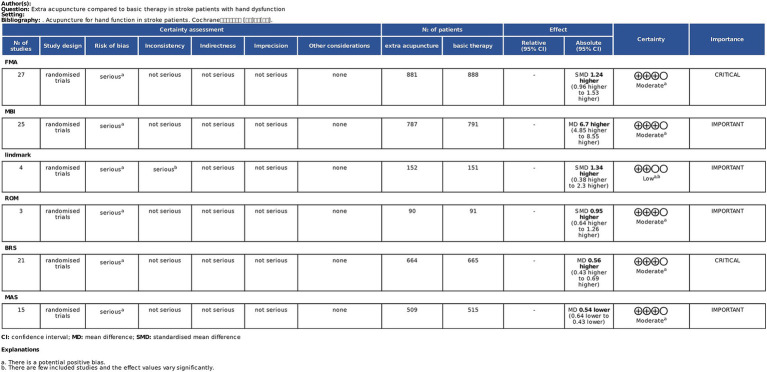
Robustness of the synthesized results.

Sensitivity analysis was conducted using RevMan 5.4. Funnel plots for all outcome measures were generated, with symmetrical distributions suggesting low publication bias (Figure 10). To evaluate the stability of the results, sensitivity analyses were performed by sequentially excluding individual studies. The pooled effect sizes exhibited minimal fluctuation, confirming the robustness of the findings. GRADEpro was used to assess the certainty in the body of evidence for each outcome evaluated. The results indicated that the overall confidence level of the evidence was moderate (Figure 11).

## 4 Discussion

In this study, we enhanced the credibility and quality of the research by implementing rigorous experimental controls, including standardized protocols and strict adherence to pre-defined inclusion/exclusion criteria. These measures minimized potential confounding variables, ensuring that the observed therapeutic effects could be attributed specifically to acupuncture. To our knowledge, this is the first investigation to evaluate the efficacy of acupuncture for post-stroke hand dysfunction using a comprehensive rehabilitation assessment framework to quantify functional outcomes. This study systematically evaluated the safety and efficacy of acupuncture in improving hand function in stroke patients through a meta-analysis of 42 trials. The results demonstrated that acupuncture significantly improved hand muscle tone, ROM, and functional performance, while also enhancing activities of daily living. Moreover, acupuncture exhibited favorable tolerability, high compliance, and a low incidence of adverse events, with no severe complications reported. We compared TA methods with EA. Notably, EA demonstrated superior efficacy to TA in enhancing BRS stages, with lower heterogeneity observed in improving FMA scores.

It is noteworthy that blinding implementation in acupuncture research remains a critical methodological challenge. Our meta-analysis indicates that most studies fail to rigorously adhere to blinding standards, a limitation rooted in two intrinsic characteristics of these therapies. First, successful acupuncture requires inducing *deqi* (a characteristic compound sensation including pain, numbness, or swelling through precise acupoint stimulation), which inherently conflicts with blinding. Since *deqi* is both a prerequisite for therapeutic efficacy and a subjective patient experience, patients often recognize treatment authenticity during the intervention, thereby compromising outcome objectivity. Second, an inherent paradox exists in operator blinding: while using the same practitioner for real and sham interventions ensures procedural consistency, it risks unblinding, as practitioners may infer group allocation based on acupoint selection or manipulation techniques. Conversely, employing different practitioners introduces variability in manipulation skills, potentially biasing treatment effects. Notably, omitting sham acupuncture controls further increases unblinding risks. These dual challenges—patient-perceived *deqi* and operator-dependent efficacy—suggest that overemphasizing blinding as the sole criterion for assessing methodological quality in acupuncture research may be inappropriate.

As an indispensable part of traditional Chinese medicine, acupuncture exerts multimodal therapeutic effects in stroke rehabilitation through molecular and neurophysiological mechanisms. At the molecular level, EA inhibits cellular pyroptosis in cerebral ischemia/reperfusion injury models via PI3K/AKT/mTOR signaling (53), while concurrently mitigating neuroinflammation through cytokine regulation (54, 55). These neuroprotective effects synergize with angiogenesis promotion via miR-7 suppression (56) preserving peri-infarct neuronal viability. Functionally, acupuncture-induced neuroplasticity manifests as large-scale activation of sensorimotor networks and thalamic regions (57), with contralateral stimulation further modulating regional homogeneity in critical brain areas (58). Such plasticity facilitates neural circuit rewiring through enhanced neurogenesis and synaptic sprouting (59), directly supporting recovery of fine motor control—a prerequisite for hand function rehabilitation requiring precise cortical mapping.

Clinically, the therapeutic superiority of EA over TA may originate from three interlinked mechanistic advantages: first, electrical stimulation ensures the activation of corticospinal pathway (60), promoting synaptic remodeling and facilitating compensatory neural response generation post nerve injury; second, enhanced functional connectivity provides sustained neuromodulator effects (61, 62) particularly advantageous for patients with sensorimotor integration deficits; finally, through quantifiable parameters (e.g., frequency, intensity) reproducibly, EA avoids the dependence of manual acupuncture techniques on the operator, thus achieving personalized and precise solutions that are difficult to achieve with TA.

Neuroplasticity mechanisms and our findings clarify the significant heterogeneity in TA efficacy. This variability primarily stems from operator-dependent factors, including (1) acupoint selection criteria (e.g., anatomy-guided vs. meridian-based approaches) and (2) manipulation techniques (e.g., needle depth, angular dynamics). In resource-limited clinical settings where operator skill levels vary, EA demonstrates superior reproducibility through its parameter-driven approach. Healthcare systems in underserved areas (such as rural regions or primary care facilities in developing countries) are often confronted with multifaceted challenges, including shortages of medical equipment and insufficient healthcare professionals. As a combination of TA and electrical stimulation, EA provides a simple, safe and effective rehabilitation method due to its standardized scheme, cost-effectiveness and evidence-based treatment benefits, which is worth popularizing.

This study has several limitations. First, while we intentionally avoided overemphasizing blinding procedures for doctors and patients, most included studies failed to report whether outcome assessors or statistical analysts were blinded, nor did they describe allocation concealment methods. These reporting gaps may introduce bias risks, particularly for outcomes involving subjective measurements. Second, in the TA group, variability in acupoint selection criteria and acupuncture techniques resulted in high heterogeneity, preventing subgroup analyses to assess the specific effects of different acupuncture methods or acupoint choices on post-stroke hand dysfunction. Future trials should adopt standardized protocols and incorporate studies from diverse countries/regions to better compare and evaluate the efficacy of distinct acupuncture approaches.

## 5 Conclusion

Our study confirms the efficacy of acupuncture in alleviating post-stroke hand dysfunction, with electroacupuncture demonstrating comparative advantages over traditional methods in enhancing motor function recovery. Despite consistent reports of positive outcomes across existing studies, critical methodological limitations—particularly the inadequate implementation of allocation concealment and blinding—raise concerns about the validity of current evidence. To strengthen clinical applicability, future research must prioritize rigorous trial designs, standardized efficacy assessments, and systematic exploration of optimal acupoint combinations and stimulation parameters. These efforts will facilitate the development of evidence-based protocols for post-stroke rehabilitation.

## Data Availability

Publicly available datasets were analyzed in this study. This data can be found: all data were extracted from published studies cited in the manuscript. Summarized results are presented in the text, and raw data can be obtained by contacting the corresponding authors of the original publications.

## Supplementary material

SUPPLEMENTARY FILE 1 The complete search strategies for all databases.

SUPPLEMENTARY FILE 2 Bonferroni correction of BRS.

SUPPLEMENTARY FILE 3 List of abbreviations.
