# Supplementary material for: Efficacy of different acupuncture therapies on hand dysfunction in post-stroke patients: a systematic review and meta-analysis
Source: Front Neurol. 2025 May 22;16:1589874. doi: 10.3389/fneur.2025.1589874 (PMC12139417; doi:10.3389/fneur.2025.1589874)
Supplement: SUPPLEMENTARY FILE 1 — The complete search strategies for all databases. [file Supplementary_file_1.docx]

Pubmed

(((((Stroke[MeSH Terms]) OR (stroke[Title/Abstract])) OR (cerebrovascular accident[Title/Abstract])) OR (cerebral stroke[Title/Abstract])) OR (CVA[Title/Abstract])) AND (((Acupuncture Treatment[MeSH Terms]) OR (Therapy, Acupuncture[MeSH Terms])) OR (Acupuncture[Title/Abstract])) AND ((hand[MeSH Terms]) OR (hand[Title/Abstract]))

Web of science

(MH=(Stroke) OR TS=(stroke) OR AB=(stroke) OR TS=(cerebrovascular accident) OR AB=(cerebrovascular accident) OR TS=(cerebral stroke) OR AB=(cerebral stroke) OR TS=(CVA) OR AB=(CVA)) AND (MH=(Acupuncture Therapy) OR TS=(Acupuncture) OR AB=(Acupuncture)) AND (MH=(Hand) OR TS=(Hand) OR AB=(Hand))

Embase

#1 'cerebrovascular accident'/exp OR 'cerebrovascular accident':ab,kw,ti OR 'stroke':ab,kw,ti OR 'cerebral stroke':ab,kw,ti OR 'cva':ab,kw,ti
#2

'acupuncture'/exp OR 'acupuncture':ab,kw,ti
#3

'hand'/exp OR 'hand':ab,kw,ti

#1 AND #2 AND #3

Cochrane Library

#1 (stroke):ti,ab,kw

#2 MeSH descriptor: [Stroke] explode all trees
#3 (cerebrovascular accident):ti,ab,kw OR (cerebral stroke):ti,ab,kw OR (CVA):ti,ab,kw

#4 #1 OR #2 OR #3

#5 MeSH descriptor: [Acupuncture Therapy] explode all trees

#6 (Acupuncture):ti,ab,kw

#7 #5 OR #6
#8 MeSH descriptor: [Hand] explode all trees
#9 (hand):ti,ab,kw
#10 #8 OR #9
#11 #4 AND #7 AND #10

CNKI

(TI ='中风' OR TI='卒中' OR SU='卒中' OR SU='中风' OR AB='卒中' OR AB='中风') AND (TI ='针灸' OR TI='针刺' OR SU='针刺' OR SU='针灸' OR AB='针刺' OR AB='针灸') AND (TI ='手' OR SU='手' OR AB='手')

Wanfang

(主题:(针灸 OR 针刺) OR 题名或关键词:(针灸 OR 针刺) OR 摘要:(针灸 OR 针刺)) AND (主题:(中风 OR 卒中) OR 题名或关键词:(中风 OR 卒中) OR 摘要:(中风 OR 卒中)) AND (主题:(手) OR 题名或关键词:(手) OR 摘要:(手))

VIP

(U=(针灸 OR 针刺) OR T=(针灸 OR 针刺) OR R=(针灸 OR 针刺)) AND (U=(中风 OR 卒中) OR T=(中风 OR 卒中) OR R=(中风 OR 卒中)) AND (U=(手) OR T=(手) OR R=(手))

CBM

("针灸疗法"[不加权:扩展] OR "针灸"[常用字段:智能] AND "针刺"[常用字段:智能]) AND ("卒中"[不加权:扩展] OR "卒中"[常用字段:智能] AND "中风"[常用字段:智能]) AND ("手"[不加权:扩展] OR "手"[常用字段:智能])
