# Supplementary figures and images for: Efficacy of different acupuncture therapies on hand dysfunction in post-stroke patients: a systematic review and meta-analysis
Source: Front Neurol. 2025 May 22;16:1589874. doi: 10.3389/fneur.2025.1589874 (PMC12139417; doi:10.3389/fneur.2025.1589874)

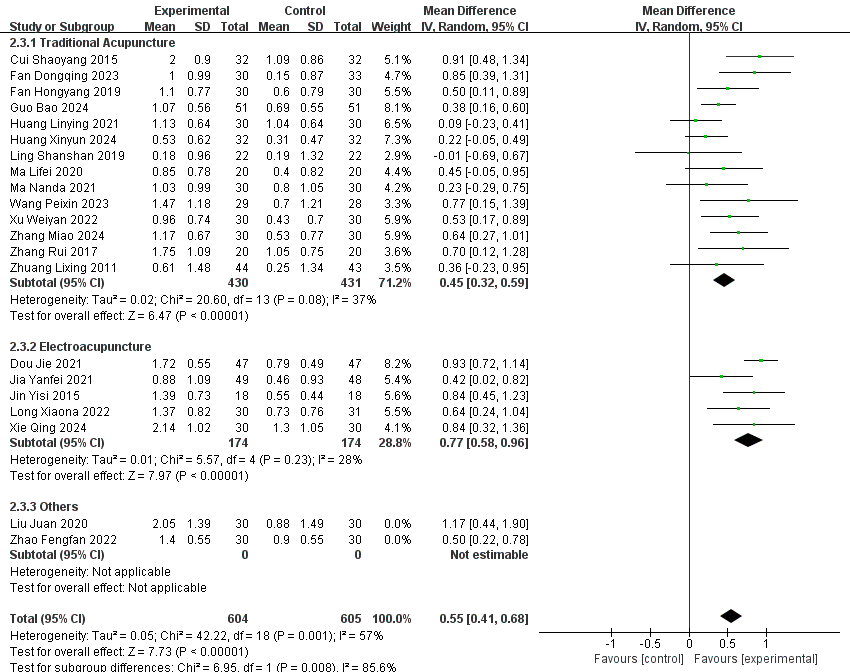

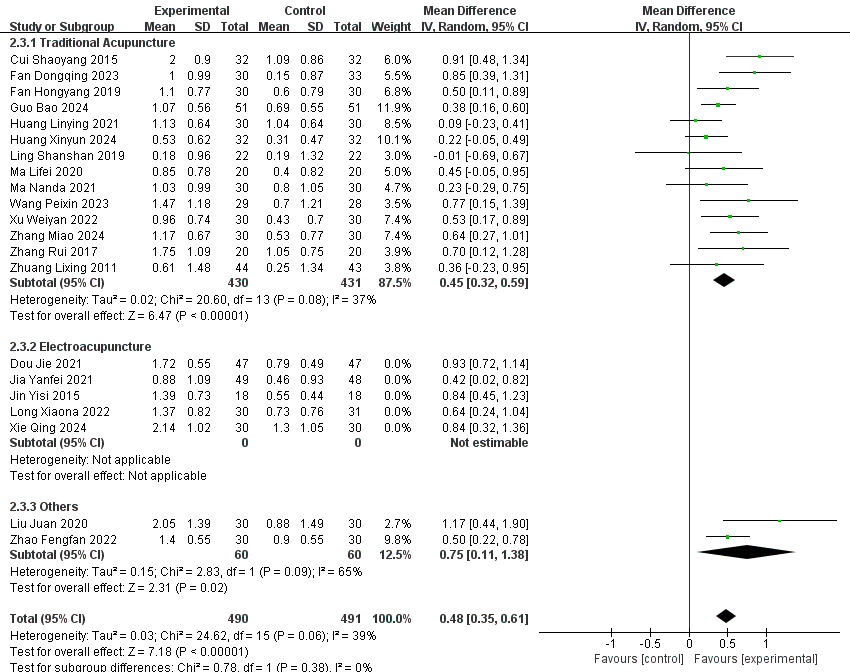

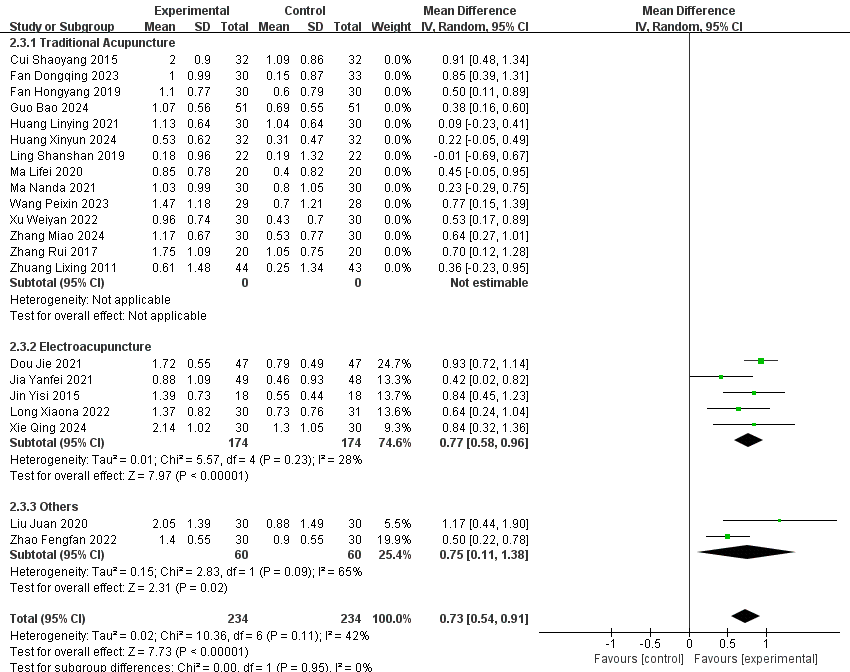

Supplement: SUPPLEMENTARY FILE 2 — Bonferroni correction of BRS. [file Supplementary_file_2.docx]
