# Supplementary material for: Efficacy of different acupuncture therapies on hand dysfunction in post-stroke patients: a systematic review and meta-analysis
Source: Front Neurol. 2025 May 22;16:1589874. doi: 10.3389/fneur.2025.1589874 (PMC12139417; doi:10.3389/fneur.2025.1589874)
Supplement: SUPPLEMENTARY FILE 3 — List of abbreviations. [file Supplementary_file_3.docx]

EA: electroacupuncture

TA: traditional acupuncture

BRS：Brunnstrom Recovery Stage

FMA：Fugl-Meyer Assessment

MAS：Modified Ashworth Scale

ROM：Range of Motion

MMT：Manual Muscle Testing

MBI：Modified Barthel Index

RCT：Randomized Controlled Trial

CNKI：China National Knowledge Infrastructure

VIP：Chongqing VIP Chinese Scientific Journals Database

CBM：China Biology Medicine

ROB：Risk of Bias
